# Health-Related Outcomes of Stand-Up Paddle Across Diverse Populations: A Scoping Review

**DOI:** 10.3390/healthcare14213705

**Published:** 2026-11-01

**Authors:** Teresa Bento, Henrique Frazão, Nuno Couto, João Freitas

**Affiliations:** 1 Sport Sciences School of Rio Maior, Santarém Polytechnic University (ESDRM-UPSantarém), 2040-413 Rio Maior, Portugal; frazao@esdrm.ipsantarem.pt (H.F.); ncouto@esdrm.ipsantarem.pt (N.C.); joaofreitas@esdrm.ipsantarem.pt (J.F.); 2 Research Center in Sports Sciences, Health Sciences, and Human Development (CIDESD), 2040-413 Rio Maior, Portugal

**Keywords:** water sports, rehabilitation, health promotion, blue spaces, nature-based interventions

## Abstract

**Highlights:**

**What are the main findings?**

- SUP may provide integrated cardiovascular, musculoskeletal, and psychological health-related outcomes across diverse populations.
- Current research remains limited by methodological heterogeneity, small samples, and the absence of randomized controlled trials, highlighting priorities for future research.

**What is the implication of the main finding?**

- The results from the present study identify research gaps in the effects of regular participation in SUP and the underlying mechanisms that explain effects across multiple health domains.

**Abstract:**

Background/Objectives: Stand-up paddle (SUP) is a rapidly growing water-based activity with potential physical- and mental-health-related outcomes. Although research on SUP has increased in recent years, the evidence remains dispersed across different populations and health outcomes, and no scoping review has specifically mapped its health-related effects. This scoping review aims to map the available evidence on the health-related outcomes of SUP across diverse populations and identify key directions for future research. Methods: A scoping review was conducted following the JBI methodology and reported according to the PRISMA-ScR guidelines. Searches were performed in PubMed, Web of Science, and RCAAP, including published and gray literature. Studies investigating the health-related effects of stand-up paddleboarding across any population were eligible. Data were summarized descriptively by main topics and health-related outcomes. Results: Eleven studies published between 2013 and 2026 met the inclusion criteria. The evidence was mapped into four thematic areas: physical conditioning and cardiovascular health, musculoskeletal strength and balance, mental health and psychological well-being, and long-term impact and mindset shifts. The available evidence was limited by small sample sizes, heterogeneous methodologies, and the absence of randomized controlled trials. Conclusions: The results from the present study identify research gaps on the health-related outcomes of regular participation in SUP and the underlying mechanisms that explain effects across multiple health domains. Finally, systematic reviews on the topic are recommended only if more robust and broader databases and search tools are used.

## 1. Introduction

Stand-Up Paddle (SUP) is a water-based physical activity in which a participant stands upright on a board and uses a single long-bladed paddle for propulsion [1]. SUP combines elements from different water sports such as surfing, kayaking, and rowing [2], and is regarded as among the most rapidly expanding sports and leisure pursuits globally [3].

The popularity of SUP has been attributed to its accessibility for all ages and skill levels, as it is relatively easy to learn, has low impact on the joints, and can be practiced in diverse aquatic settings [4].

Emerging evidence suggests that SUP may improve several aspects of physical health, including cardiovascular fitness, musculoskeletal function, and metabolic health. Specifically, SUP performed at a determined cadence meets the criteria for moderate-intensity exercise [5]. Moreover, elite SUP athletes exhibit maximal oxygen uptake [1,6], comparable to that of participants in other upper-limb-dominant water sports, and they also show favorable lipid profiles, including higher HDL levels and lower triglycerides [1]. In addition, interventions in previously untrained individuals resulted in significant improvements in cardiovascular health, core strength, and quality of life [2].

In addition to physical outcomes, participation in blue-space activities has been associated with improvements in psychosocial well-being [7], and several interventions incorporating physical activities on or near water (like surfing and SUP) have been shown to improve mental health and overall well-being across diverse populations [7,8,9,10,11]. On this matter, a scoping review by Cugusi in 2023 [12] appraised the health effects of outdoor water sport activities for chronic disease populations, suggesting beneficial effects of these activities in such populations.

Although research on SUP has expanded over the past decade, the evidence remains fragmented across different disciplines and study populations. Two previous reviews synthesized the available literature from the past decade [13,14], summarizing research across physiology, biomechanics, anthropometry, injury-related factors, pedagogical approaches, and equipment-related safety. According to these reviews, most studies have focused on applying several disciplines to performance and competitive contexts, with relatively few examining health outcomes or other populations beyond athletes.

No review has specifically mapped the health-related outcomes of SUP across different populations; therefore, this scoping review aims to map the available evidence on the health effects of SUP across diverse populations and identify key directions for future research.

### Review Question

The primary question of this scoping review is as follows: “What evidence exists regarding the health-related outcomes of Stand-Up Paddle?” The primary question can be expanded into two secondary questions: 1. “How has research on the health-related outcomes of Stand-Up Paddle been approached across different disciplines?”; 2. “What are the health-related effects of Stand-Up Paddle for specific populations?”

## 2. Materials and Methods

### 2.1. Protocol and Registration

This scoping review followed the JBI methodological framework [15,16,17] and was reported in line with the PRISMA-ScR guideline and checklist [18]. The protocol was registered on the Open Science Framework (OSF) (registration: osf.io/v7r5c).

### 2.2. Eligibility Criteria

#### 2.2.1. Inclusion Criteria

Studies involving participants of any age or skill level (including elite athletes and recreational paddlers), sedentary or active, or any specific groups (with any known disease or condition) were included. Studies were eligible if they:

- Investigate SUP as the primary intervention or activity;
- Reported at least one health-related outcome (such as physical fitness, cardiovascular health, balance, strength, mental health, quality of life);
- Studies addressing injury prevention were included only when injury prevention was examined from a public health perspective rather than a sport performance or competition perspective;
- Be focused on SUP specifically, and not on other paddle sports (kayaking, canoeing, traditional surfing). Studies including multiple sports interventions were eligible only if they provided separate SUP-specific results;

Studies conducted in field or laboratory settings were eligible. Publications written in English or Portuguese were included.

#### 2.2.2. Exclusion Criteria

Studies were excluded if they focused exclusively on SUP-related injuries, performance, biomechanics, or physiological outcomes primarily related to sport performance, or did not report relevant data.

### 2.3. Information Sources and Searches

Because evidence on SUP is limited, both published and unpublished literature were considered. All research methodologies, qualitative reviews, quantitative reviews, and literature reviews were included if they met the inclusion criteria.

Based on search terms from previous reviews and a prior exploratory search of available resources, searches were conducted in the following databases: PubMed, Web of Science (Core Collection), and Institutional Scientific Repositories (RCAAP) between 1 February and 15 July 2026, with no time-limit restrictions. The search strategy combined two concepts: (1) stand-up paddle and (2) health-related outcomes, considering the following terms and combinations: “standup paddle”; “standup paddleboarding”; “standup paddling”; “SUP”; “standup paddle and health”. The strategy was first developed for PubMed and subsequently adapted to the syntax and indexing of the remaining databases. Searches in RCAAP also included Portuguese translations of these terms to increase the likelihood of identifying academic works done on the topic. The reference list of previous articles was also searched. A detailed search strategy can be found in Appendix A.

### 2.4. Selection of Sources of Evidence

This scoping review followed a three-stage search process. In the first stage, an initial exploratory search was carried out in the selected databases using predefined keyword combinations. In the second stage, the terminology identified in relevant titles, abstracts, and indexing terms was then examined to refine the search, and at the third stage of the process, the complete search strategy was finalized.

All retrieved records were exported to EndNote for organization and removal of duplicates. The screening process was performed independently by two reviewers and consisted of two sequential steps. First, titles and abstracts were evaluated according to the established inclusion criteria. After that, records that could not be excluded based on title and abstract proceeded to full-text screening.

Subsequently, the full-text articles were reviewed against the inclusion criteria. When full texts were not available, the corresponding authors were contacted via email or ResearchGate. A follow-up was done after one week if no response was received. Two independent screeners performed the searches. Divergent screener decisions were resolved by consensus. When consensus was not reached, a third reviewer was added to the discussion.

### 2.5. Data Charting Process

Data were extracted by one reviewer and subsequently checked by a second reviewer. Any discrepancies identified during this process were resolved through discussion. The extracted data were compiled in an Excel sheet and included the following:

- Author name(s), title, year of publication, country of publication; specific aspect studied (e.g., physiological responses, biomechanics, balance/stability, cardiovascular fitness, rehabilitation applications);
- Sample size and demographics (age, gender, Body Mass Index—BMI), skill level (elite/competitive, recreational, beginner), health status (healthy individuals, rehabilitation patients, specific conditions);
- Health outcomes;
- Source type.

### 2.6. Synthesis of Results

Frequencies and descriptive statistics were used to summarize study characteristics. Findings were narratively synthesized and organized by main topics and health-related outcomes.

## 3. Results

### 3.1. Selection of Sources of Evidence

A total of 236 records were identified through database searching and 2 through other sources. After removing duplicates, 89 records were screened by title and abstract. Of these, 79 were excluded for reasons such as “injuries related”, “not related/no relevant data”, and focusing on “performance, physiology, or biomechanics” not related to health. Ultimately, 11 sources of evidence were included in this scoping review. The flow diagram, representing the selection process, is presented in Figure 1.

### 3.2. Characteristics of Sources of Evidence

The characteristics of the 11 included studies were charted to reflect their geographic origin, population demographics, and the specific aspects of SUP health-related outcomes studied. A summary of the study characteristics is presented in Table 1. 

The studies were published between 2013 and 2026 and were predominantly conducted in Australia, followed by Brazil, Denmark and the UK, and then Austria and Portugal, as shown in Figure 2.

Sample sizes ranged from less than 10 participants [4,5,11,19,20,21,22], with 2 of them being case studies [4,19]. The studies with the largest samples included 45 [1] and 60 [3] participants.

Adults constituted the primary population, with two other studies including children and/or adolescents [3,22], and only one included older people [23]. Most studies included both males and females in their samples, but three studies included only female samples [11,20,23], and one included only male participants [22].

**Table 1:** Summary of characteristics of the included studies.

Author, Year	Objective	Studied Variables	Health Outcomes
Ruess et al. (2013) [3]	To prove that SUP is a suitable endurance training for amateurs and that it improves balance and stability.	Physiological responses (heart rate); balance/stability training	Heart rate of beginners stays in the aerobic zone, making SUP suitable for endurance training. Significant improvement in balance stability.
Schram et al. (2016a) [2]	To conduct a training intervention on previously untrained individuals to ascertain the potential effects of SUP on various health parameters.	Physiological (aero-bic/anaerobic fitness), musculoskeletal (core strength, balance), and psychological (QoL) effects	Significant improvements in aerobic (+23.6%) and anaerobic (+42%) fitness, multidirectional core strength, and quality of life (physical/psychological). No significant change in balance.
Schram et al. (2016b) [1]	To profile elite and recreational SUP participants regarding anthropometric, physiological, and musculoskeletal measurements compared to sedentary individuals.	Physiological (fitness, lipids), anthropometric, and musculoskeletal profiling	Elite paddlers possess superior aerobic/anaerobic power, better static/dynamic postural control, higher trunk endurance, and more favorable lipid profiles (higher HDL, lower triglycerides).
Schram et al. (2017) [4]	To document the long-term (one-year) effects of participation in SUP on fitness, trunk endurance, and quality of life through a case study of two participants.	Physiological, musculoskeletal, and psychological effects	Maintenance of health gains: reduced body mass, BMI, and body fat; improved trunk endurance and aerobic fitness; significant, sustained increases in all QoL domains.
Caetano et al. (2018) [19]	To evaluate changes in body composition and somatotype resulting from eight weeks of SUP practice in sedentary individuals.	Changes in body composition and somatotype	Positive changes in body composition, including decreased BMI and body fat percentage, and improved somatotype results.
Osti et al. (2018) [23]	To evaluate the effect of on-water SUP training on upright postural control and balance stability in healthy older adults.	Upright postural control (balance stability)	SUP practice significantly reduced body sway (improved balance) in challenging postures, even with eyes closed, compared to walking control.
Willmott et al. (2020) [5]	To quantify physiological and perceptual responses of SUP at various stroke rates in laboratory and field settings to determine if it meets moderate-intensity exercise criteria.	Physiological responses (HR, EE, METs) and perceptual responses (RPE, enjoyment)	Linear increases in HR and energy expenditure with stroke rate; SUP at ≥20 strokes/min meets criteria for moderate-intensity exercise (3.0–5.9 METs). High ratings of enjoyment.
Sparre & Østergaard (2023) [11]	To explore the experiences of people living with mental disorders practicing SUP and to determine if and how these experiences influence their mental health.	Qualitative impact of physical bodily experiences on mental health (mind–body connection, flow)	Improved mental health; reduced rumination; feelings of presence, flow, and freedom; increased self-confidence and social integration.
Østergaard et al. (2024) [20]	To explore the long-term (up to 3.5 years) experiences and mental well-being impact of participating in blue space SUP activities for individuals with mental disorders	Long-term (up to 3.5 years) impact of blue space activities on mental well-being	Life-changing impact on mental/social life; outward mindset shift; increased confidence and self-efficacy; sustained breaks from rumination and connection to nature/peers.
Curado (2025) [21]	To analyze the impact of a 6-week SUP intervention on the mental health, functional physical fitness, and body composition of people with Intellectual and Developmental Disability (IDD).	Impact on mental health (anxiety/depression) and functional physical fitness (balance, strength, agility)	Significant decrease in anxiety symptoms and significant improvement in dynamic balance; no significant changes in depression or strength.
Hornsby et al. (2026) [22]	To explore the experiences of vulnerable young people participating in an urban SUP therapy intervention and its influence on their health and well-being.	Qualitative experiences of vulnerable youth in SUP therapy (mental health/well-being)	Feelings of calmness and respite from worries; pride in mastering skills (standing); increased self-confidence/self-esteem; reduced social anxiety and anger.

The included studies involved diverse populations, ranging from healthy individuals [1,2,3,4,5,19,23], vulnerable youth, and individuals with mental disorders or intellectual and developmental disabilities [11,20,21,22], to sedentary adults and elite athletes, and older adults. The experience level in SUP included mostly beginners, with intermediate and experienced individuals in other cases [1,3,20]. Characteristics of the populations involved in the studies are mapped in Figure 3.

### 3.3. Synthesis of Results

In order to answer the primary question of this scoping review: “What evidence exists regarding the health-related outcomes of Stand-Up Paddle?” The findings were organized descriptively into broad areas based on the topics and health-related outcomes addressed in the included studies. These categories were used to structure the presentation of the results and were not derived through formal coding or qualitative content analysis.

Main topics and health-related outcomes addressed in the included studies are presented in Figure 4.

Regarding study design, most included studies employed quasi-experimental or non-randomized intervention designs (n = 3) or qualitative methodologies (n = 3), followed by case studies (n = 2) and physiological or laboratory-based studies (n = 2). Only one study used a cross-sectional observational design.

The types of evidence available ranged from interventional training studies and cross-sectional profiling to long-term qualitative follow-ups and pilot project reports. None of the studies was a randomized controlled trial (RCT). Together, these findings indicate that the current evidence base remains exploratory and is largely derived from non-randomized designs.

## 4. Discussion

This review mapped the available evidence on the health -related outcomes of SUP across different populations and health domains.

### 4.1. Summary of Evidence

This scoping review mapped the available evidence on the health-related outcomes of SUP across diverse populations and health domains. Overall, the studies reported that SUP is a low-impact, accessible activity with potential effects for both physical and psychological health. The available evidence spans exercise physiology, rehabilitation, psychology, and public health, highlighting the multidisciplinary nature of current SUP research.

However, despite these promising findings, the available evidence remains limited. Most studies included small samples, only one reported longitudinal follow-up [4,20], and none employed randomized controlled trial (RCT) designs. Consequently, the findings should be interpreted as indicative rather than conclusive.

To answer the primary question of this scoping review: “What evidence exists regarding the health-related outcomes of Stand-Up Paddle?” Despite acknowledged methodological limitations, the analyzed evidence suggests that the “Physical Conditioning and Cardiovascular Health” outcomes of SUP indicate that this activity may function as an aerobic workout and endurance training tool. Interventions involving previously sedentary individuals demonstrated improvements in aerobic and anaerobic fitness [2], and physiological quantification may indicate that SUP at stroke rates of 20 per minute or higher meets the criteria for moderate-intensity exercise (3.0–5.9 METs) [5]. Furthermore, the activity appears to promote positive body composition changes, including reductions in weight, BMI, and body fat percentage [1,4,19], and favorable lipid profiles (high HDL, low LDL/triglycerides) [1]. The high rate of adherence to this activity was observed in several studies [2,4,19,21,23], as seen in other water sports [12], which may be relevant for diseased populations that, more than others, benefit from continued participation in physical activity.

Regarding the “Musculoskeletal Strength and Balance” health outcomes of SUP, studies reported the development of multidirectional trunk endurance and core strength [1,2]. Among individuals with IDD, studies also showed an association with improvements in dynamic balance [21].

As for the “Mental Health and Psychological Well-being” and the “Long-term Impact and Mindset Shifts” health outcomes of SUP, both qualitative and quantitative research indicated SUP as a potential mental health intervention [11,20,21,22]. Multiple interacting mechanisms may explain the improvements in psychological well-being reported across the included studies [12,20]. Beyond short-term improvements in mood, qualitative evidence suggests that sustained participation may lead to broader psychological changes, including greater social connectedness and a shift from a diagnosis-centered perspective toward a more outward-focused engagement with everyday life [20]. Furthermore, in people with IDD, SUP seems to contribute to reducing anxiety [21]. The “colleagueship” and sense of belonging found in group-based SUP programs seemed to serve as critical components for social integration and mental well-being [11,20].

Research gaps were identified. Collectively, the findings from the health-related outcomes of SUP reported in this scoping review raised questions to potentially be addressed in future research, such as: “May regular SUP participation positively influence: (a) cardiovascular risk profiles; (b) fall prevention; (c) low back pain; (d) stress?”; and “Could the interaction between physical and environmental characteristics explain why health-related outcomes are reported across multiple health domains?”. Future research should also prioritize adequately powered randomized controlled trials, standardized intervention protocols, and longer-term follow-up to establish dose–response relationships and determine the magnitude and sustainability of the SUP’s health-reported effects.

Finally, based on the available evidence, it does not seem recommendable to perform a systematic review of studies on the topic unless more robust and broader database and search tools are used.

### 4.2. Limitations

Although a comprehensive search strategy was employed, relevant studies may have been missed, particularly gray literature not indexed in the selected sources or publications in languages other than English and Portuguese. Also, the use of more robust databases could have further strengthened this review.

The available evidence also presents several methodological limitations. Most studies included small samples, and intervention studies frequently lacked independent control groups. In addition, few studies adopted longitudinal designs, limiting understanding of the long-term sustainability of the reported health-related outcomes.

Most intervention studies were conducted with clinical or vulnerable populations, which may limit the generalizability of some findings to the wider population. Furthermore, the absence of RCTs and standardized SUP training protocols limits conclusions regarding optimal dose–response relationships and clinical implementation.

Finally, the multidisciplinary nature of the literature resulted in substantial heterogeneity in study designs, populations, and outcome measures, limiting direct comparison across studies.

## 5. Conclusions

This scoping review mapped the available evidence on the health-related outcomes of SUP across diverse populations and health domains. Although the current evidence base remains limited and methodologically heterogeneous, the reported findings suggest the potential impact of SUP on cardiovascular fitness, body composition, balance, musculoskeletal function, and psychological well-being.

The results from the present study identify research gaps on the health-related outcomes of regular participation in SUP and the underlying mechanisms that explain effects across multiple health domains. Future research should prioritize adequately powered randomized controlled trials, standardized intervention protocols, and longer-term follow-up to establish dose–response relationships and determine the sustainability of the reported effects. Finally, systematic reviews on the topic are recommended only if more robust and broader database and search tools are used.

## Figures and Tables

**Figure 1:**
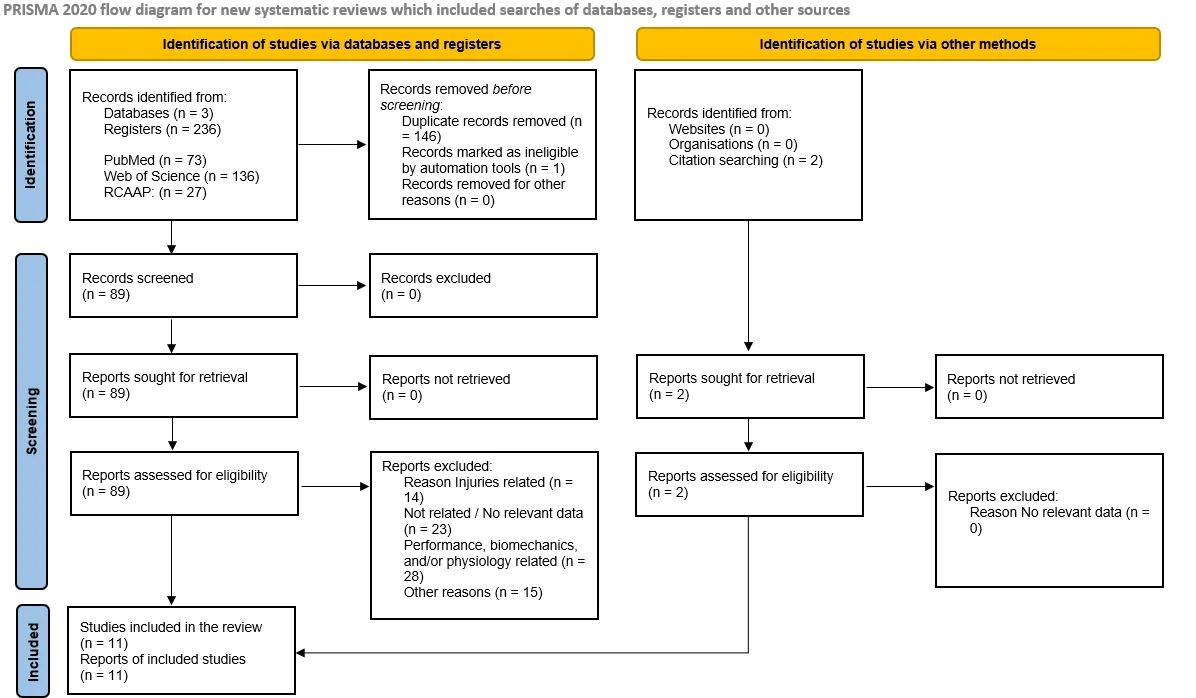
Flow diagram representing the selection of sources of evidence.

**Figure 2:**
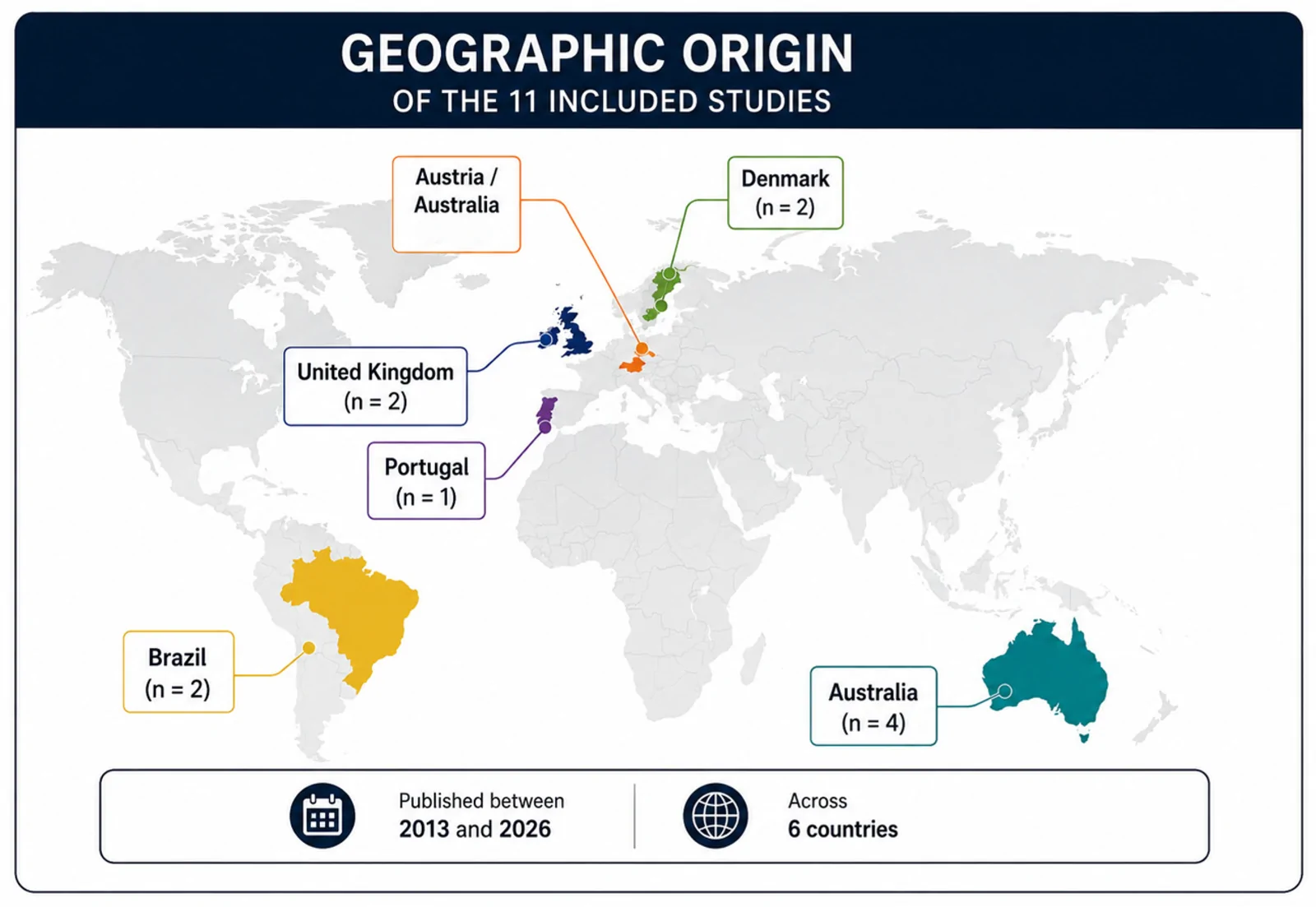
Origin of the included studies.

**Figure 3:**
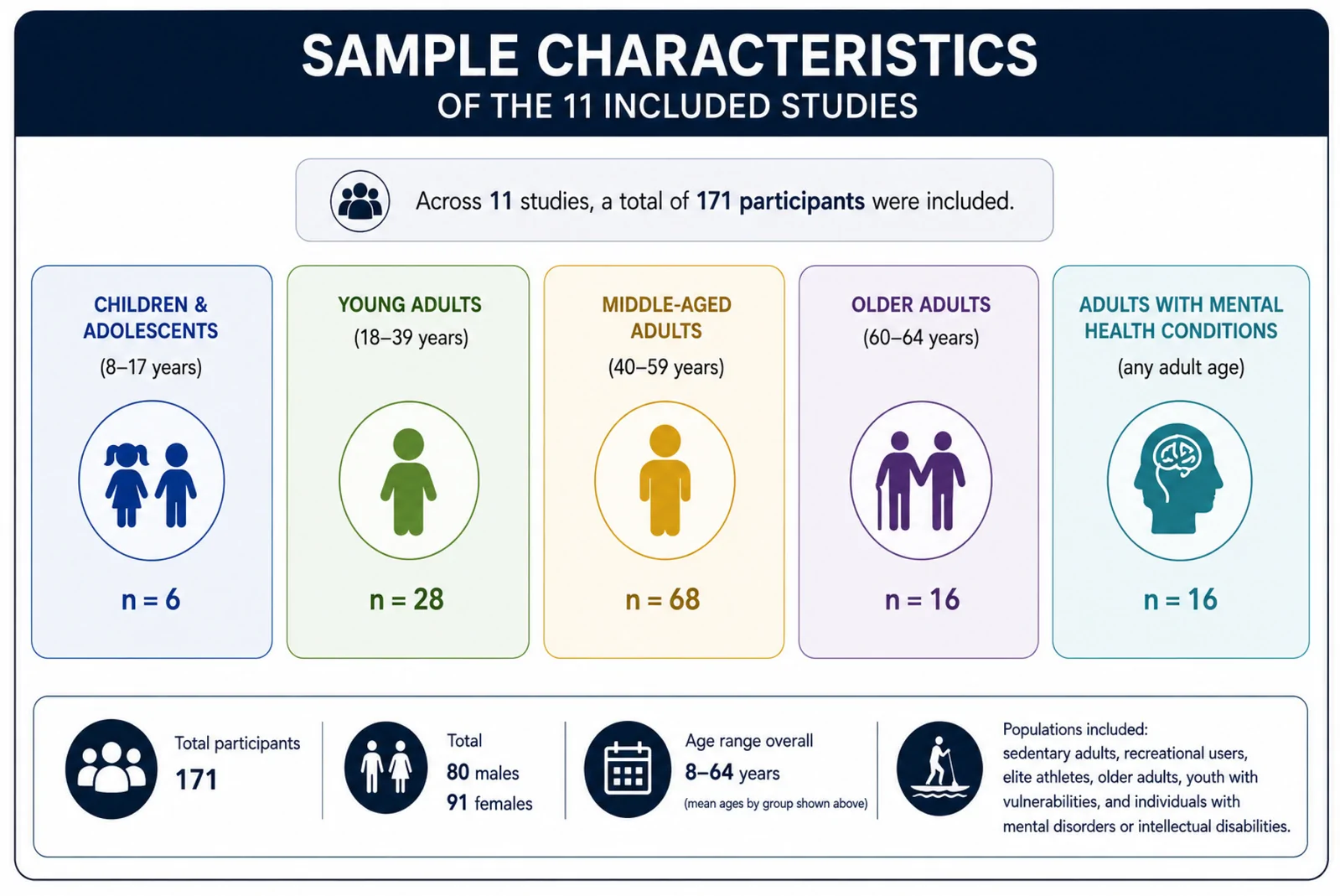
Characteristics of the population involved in the included studies. Note. Population categories may overlap, as some studies included participants from more than one population group.

**Figure 4:**
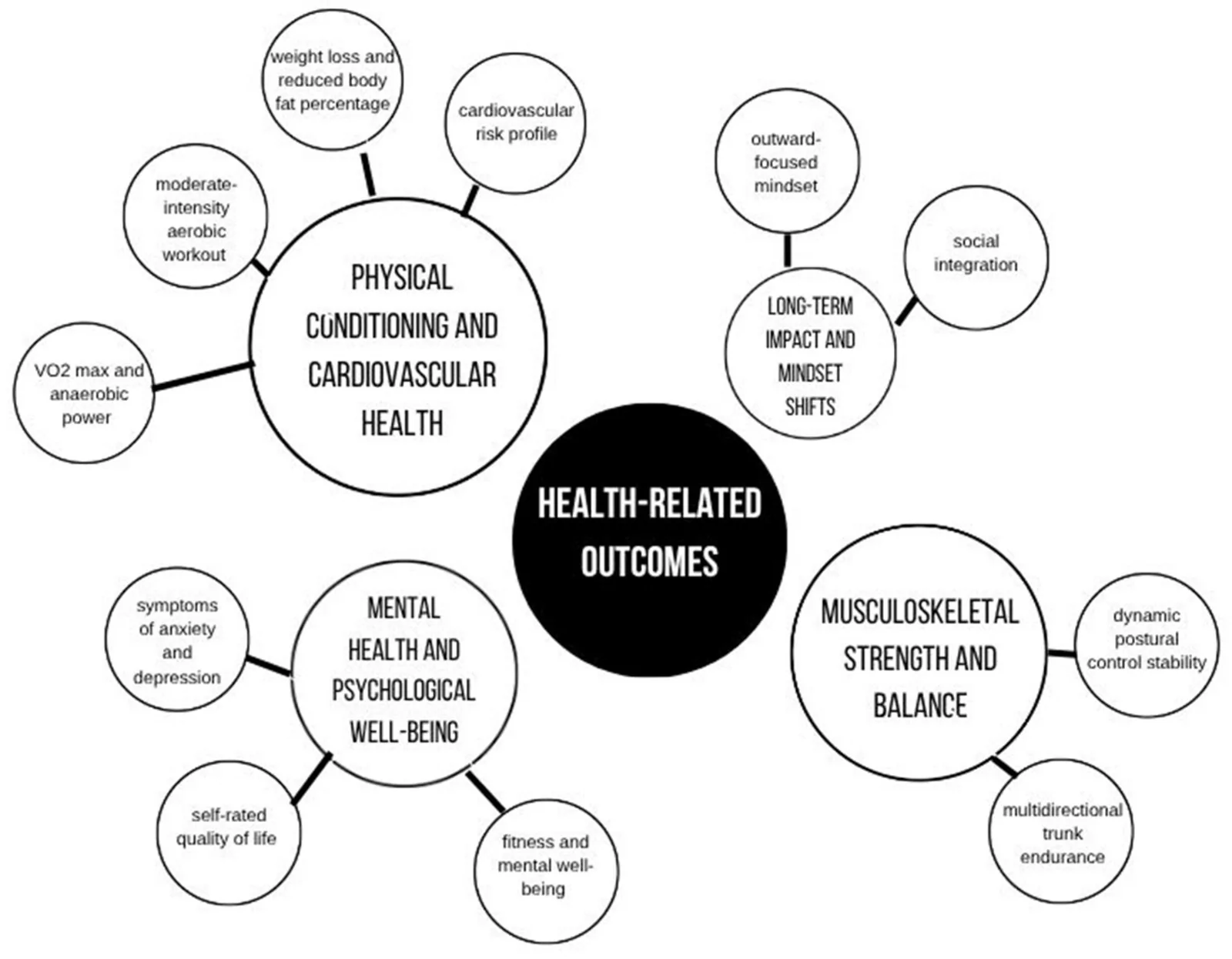
Main topics and health-related outcomes addressed in the included studies.

## Data Availability

No new data were created or analyzed in this study. Data sharing is not applicable to this article.

## Appendix A

**Table A1:** Detailed search strategy.

Concept	Search Strategy	
PubMed		
Stand-up paddleboarding	#1	“stand up paddle*”[tiab] OR “stand-up paddle*”[tiab] OR “standup paddle*”[tiab] OR “stand up paddle board*”[tiab] OR “stand-up paddle board*”[tiab] OR “standup paddle board*”[tiab] OR “stand up paddle*”[tiab] OR SUP[tiab]
Health	#2	“Health”[Mesh] OR health[tiab] OR health benefit*[tiab] OR well-being[tiab] OR wellbeing[tiab] OR quality of life[tiab] OR physical fitness[Mesh] OR fitness[tiab] OR physiolog*[tiab]
	#3	#1 AND #2
Web of Science		
Stand-up paddleboarding	#1	TS = (“stand up paddle*” OR “stand-up paddle*” OR “standup paddle*” OR “stand up paddle board*” OR “stand-up paddle board*” OR “standup paddle board*” OR SUP)
Health	#2	TS = (health OR “health benefit*” OR “well-being” OR wellbeing OR “quality of life” OR fitness OR physiolog*)
	#3	TS = (stand-up paddleboarding terms) AND TS = (health-related terms)
RCAAP		
Stand-up paddleboarding	#1	(“stand up paddle” OR “stand-up paddle” OR “standup paddle” OR “stand up paddle boarding” OR “stand-up paddle boarding” OR SUP OR “paddle surf”)
